# Chlorogenic acid inhibits glioblastoma growth through repolarizating macrophage from M2 to M1 phenotype

**DOI:** 10.1038/srep39011

**Published:** 2017-01-03

**Authors:** Nina Xue, Qin Zhou, Ming Ji, Jing Jin, Fangfang Lai, Ju Chen, Mengtian Zhang, Jing Jia, Huarong Yang, Jie Zhang, Wenbin Li, Jiandong Jiang, Xiaoguang Chen

**Affiliations:** 1 State Key Laboratory of Bioactive Substances and Functions of Natural Medicines, Institute of Materia Medica, Chinese Academy of Medical Sciences and Peking Union Medical College, Beijing 100050, China; 2 Jiuzhang Biochemical Engineering Science and Technology Development Co., Ltd., Chengdu, Sichuan 610041, China; 3 Department of Glioma, Beijing Shijitan Hospital, Capital Medical University, Beijing 100038, China

## Abstract

Glioblastoma is an aggressive tumor that is associated with distinctive infiltrating microglia/macrophages populations. Previous studies demonstrated that chlorogenic acid (5-caffeoylquinic acid, CHA), a phenolic compound with low molecular weight, has an anti-tumor effect in multiple malignant tumors. In the present study, we focused on the macrophage polarization to investigate the molecular mechanisms behind the anti-glioma response of CHA *in vitro* and *in vivo*. We found that CHA treatment increased the expression of M1 markers induced by LPS/IFNγ, including iNOS, MHC II (I-A/I-E subregions) and CD11c, and reduced the expression of M2 markers Arg and CD206 induced by IL-4, resulting in promoting the production of apoptotic-like cancer cells and inhibiting the growth of tumor cells by co-culture experiments. The activations of STAT1 and STAT6, which are two crucial signaling events in M1 and M2-polarization, were significantly promoted and suppressed by CHA in macrophages, respectively. Furthermore, In G422 xenograft mice, CHA increased the proportion of CD11c-positive M1 macrophages and decreased the distribution of CD206-positive M2 macrophages in tumor tissue, consistent with the reduction of tumor weight observed in CHA-treated mice. Overall these findings indicated CHA as a potential therapeutic approach to reduce glioma growth through promoting M1-polarized macrophage and inhibiting M2 phenotypic macrophage.

Glioblastoma multiforme (GBM) is the most aggressive pattern among brain tumors. GBM has an extremely poor prognosis status: patient survival remains less than 15 months^1^, even after surgical ablation and routine treatments with chemo- and radio-therapeutic agents. It has been widely shown that the immune microenvironment has a central role in GBM. Microglia/macrophages represent the largest tumor-infiltrating immune cell population, contributing to tumor progression and metastasis^2^. Thus, a novel research field is focused on immunotherapy-based drug discovery.

Macrophage is broadly categorized as “classically activated” pro-inflammatory M1 macrophages and “alternatively activated” anti-inflammatory M2 macrophages^3^,^4^. Macrophages can differentiate into various activation states owing to the cytokine balance in the microenvironment. M1 phenotypic macrophage activation, in response to interferon γ (IFNγ) or lipopolysaccharide (LPS), is characterized by up-regulation of inducible nitric oxide synthase (iNOS) and interleukins IL6 or IL12, and enhancement of the Th1 immune response^5^,^6^,^7^. Alternatively, M2 phenotypic macrophage activation is induced by IL-4 and IL-13 through binding to a common receptor subunit, IL-4Rα, and characterized by increased expression of arginase 1 (Arg1), IL-10 and mannose receptor. In nonmalignant or regressing tumors, the majority of tumor-associated macrophage (TAM) is M1-like, which represents pro-inflammatory activity to kill microorganisms and promote tumor lysis^5^,^7^. In malignant tumors, TAM has been shown to be of predominant in M2 phenotype, which has a pro-tumor function by secretion of proangiogenic factors^8^ and immunosuppressive cytokines^9^. Increased proportion of M2-type TAM has been associated with poor prognosis in multiple tumors including Hodgkin’s lymphoma, cholangiocarcinoma, breast carcinoma, as well as cerebral glioma^10^,^11^,^12^. Thus, several research groups focused on M2-like TAMs as a potential targets for anticancer therapies and have gained encouraging results. In addition, skewing TAM differentiation away from the M2 to M1 phenotype promoted antitumor immune responses and reduced tumor growth and metastasis in hepatocellular carcinoma (HCC), prostate cancer, breast cancer and glioblastoma^13^,^14^,^15^,^16^. Furthermore, M1-type macrophages have been reported to be associated with prolonged survival time in patients with non small-cell lung cancer (NSCLC)^17^,^18^.

Some natural compounds and therapeutic drugs have been reported to affect macrophage phenotypic differentiation to exert their antitumor effect. These compounds include the natural products zoledronic acid^19^ and emodin^20^, therapeutic monoclonal antibodies including anti-CD47^13^,^21^ and the phosphatidylserine-targeting antibody 2aG4^14^, and chemotherapy agents, doxycycline and metformin^22^,^23^. Chlorogenic acid (5-caffeoylquinic acid, CHA), the ester formed between caffeic acid and quinic acid^24^, is a phenolic compound found in the human diet, in coffee, apples, pears and in green tea. It has been shown to have several beneficial biological properties, including anti-bacterial effects, anti-inflammatory effects, and anti-oxidant effects, as well as anti-tumor activities^25^,^26^. It has been found to exert anti-inflammatory effects through NF-κB inhibition of prostaglandin E synthesis and cyclo-oxygenase-2 and by suppressing JNK/AP-1 activation^27^. It has been shown to activate calcineurin and enhance macrophage function *in vivo* and *in vitro*^28^. Furthermore, CHA has been proposed to inhibit tumor growth and angiogenesis by inhibiting tyrosinase and matrix metalloproteinase (MMP)-9^29^,^30^, or by the suppression of HIF-1α stabilizationand AKT phosphorylation^31^. Recently, published findings from pre-clinical experimental and phase I clinical studies have shown that treatment with CHA has shown therapeutic effects in breast cancer^32^, brain tumors^33^, lung cancer^34^, colon cancer^35^ and chronic myelogenous leukemia^29^. However, the molecular mechanisms behind the anti-cancer response of CHA and its effects on macrophage polarization, remains yet largely unclear and need to be clarified.

In this study, we investigate the role of CHA-mediated macrophage polarization in glioblastoma progression. We found that, *in vitro*, CHA induced LPS/IFNγ- but inhibited IL4-responsive genes through promoting STAT1 signaling and inhibiting STAT6 signaling, respectively. Thereby, CHA promoted the production of apoptotic-like cancer cells and inhibited tumor growth by affecting the interaction between macrophages and cancer cells. We also found that CHA inhibits growth of G422 glioma *in vivo*, and this effect was associated with a decrease of M2-like TAMs and recruitment of M1-like TAMs into tumor tissue.

## Results

### CHA promoted M1 polarization of macrophages induced by lipopolysaccharide (LPS) and interferon (IFN)-γ

**Figure 1:**
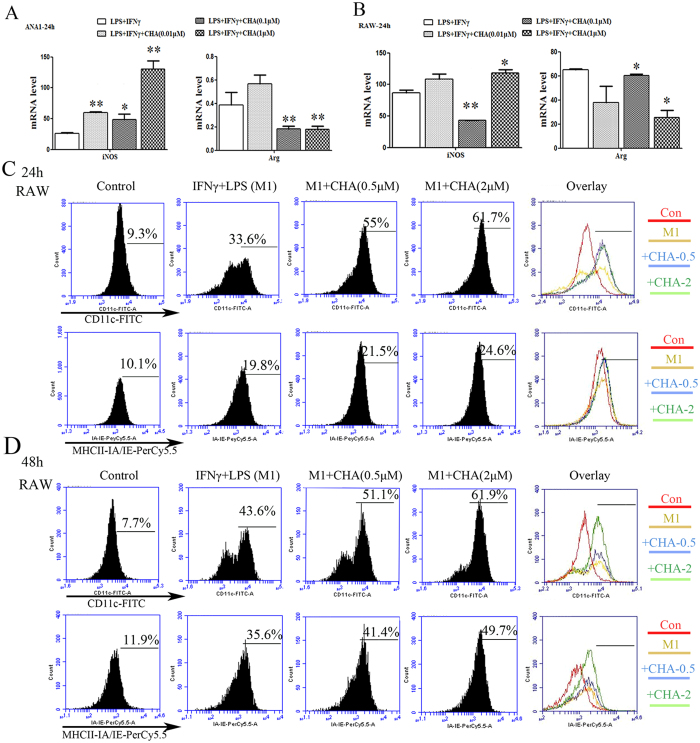
Effect of CHA on macrophage marker expression induced by lipopolysaccharide (LPS) and interferon (IFN)-γ. Macrophages were treated with LPS (10 ng/ml) and IFNγ (20 ng/ml) with or without different concentrations of CHA for 24 h or 48 h. The mRNA levels of M1-marker gene iNOS and M2-marker gene Arg in Ana-1 (**A**) and RAW264.7 cells (**B**) were measured by real-time RT-PCR. The expression of mRNAs was normalized to GAPDH. The expressions of CD11c and MHC Class II IA-IE in RAW264.7 cells for 24 h (**C**) or 48 h (**D**) were evaluated by flow cytometry. The histogram bars represent three independent experiments. The data are presented as the mean ± SD. *p-value < 0.05, **p-value < 0.01 vs. LPS/IFNγ.

We detected the effect of CHA on macrophages polarization at the concentrations of 0.5 μM to 5 μM, and the proliferation of macrophages was not affected under these doses of CHA (Supplementary Fig. 1A). Initially, the M1/M2 macrophage phenotype marker genes induced by IFNγ and LPS (M1 inducer) were analyzed using RT-PCR analysis. Treatment with IFNγ/LPS significantly up-regulated the mRNA levels of the M1 marker gene iNOS or IL12 in Ana-1 and RAW264.7 macrophages (P ≤ 0.01). Meanwhile, IFNγ/LPS treatment significantly reduced the mRNA levels of the M2 marker gene Arg1 in Ana-1 cells, but that of RAW264.7 cells was increased (P ≤ 0.01) (Supplementary Fig. 2A,B). Then, at exposure to CHA, IFNγ/LPS-induced iNOS mRNA level was further up-regulated and the Arg1 mRNA level was significantly down-regulated in Ana-1 cells in a concentration-dependent manner (P ≤ 0.05, P ≤ 0.01) (Fig. 1A). Similarly, in RAW264.7 cells, combination with 1 μM of CHA significantly increased the iNOS mRNA level and decreased the Arg1 mRNA level when compared with IFNγ/LPS-treated alnoe (P ≤ 0.05) (Fig. 1B). To further investigate the role of CHA in M1 macrophage differentiation, cell surface markers were assessed by flow cytometry. As shown in Fig. 1C and D, treatment with IFNγ/LPS increased the expression of CD11c and MHC Class II subunits IA/IE, and those were dose and time-dependently increased after combination with CHA in RAW264.7 cells, especially in the expression of CD11c. These results indicated that CHA could promote M1 phenotypic differentiation in macrophages *in vitro*.

### CHA inhibited M2 polarization of macrophages induced by interleukin (IL)-4

**Figure 2:**
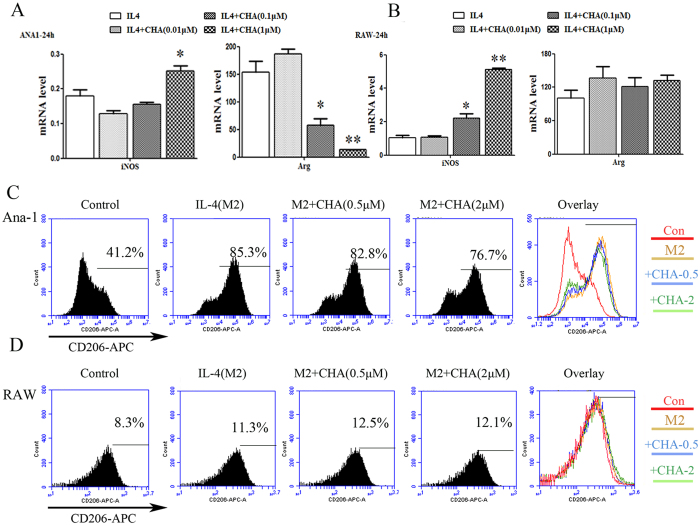
Effect of CHA on macrophage marker expression induced by interleukin (IL)-4. Ana-1 and RAW264.7 cells were treated with interleukin (IL)-4 (20 ng/ml) in the presence of DMSO or different concentrations of CHA for 24 h. The mRNA levels of M1-marker gene iNOS and M2-marker gene Arg in Ana-1 (**A**) and RAW264.7 cells (**B**) were measured by real-time RT-PCR. The expression of mRNAs was normalized to GAPDH. The expressions of CD206 in Ana-1 (**C**) and RAW264.7 cells (**D**) were evaluated by flow cytometry. The histogram bars represent three independent experiments. The data are presented as the mean ± SD. *p-value < 0.05, **p-value < 0.01 vs.IL-4.

We also analyzed the impact of CHA on M2 polarization of macrophages induced by IL-4. As shown in Supplementary Fig. 2A,B, IL-4 significantly increased the mRNA levels of the M2 marker gene Arg1 or IL10 in Ana-1 and RAW264.7 cells, and reduced the mRNA levels of the M1 marker gene iNOS in Ana-1 cells (P ≤ 0.05, P ≤ 0.01). CHA decreased the expression of the IL-4-induced Arg1 mRNA levels in a dose-dependent manner, but with a significant increase in the iNOS mRNA level at 1 μM of CHA in IL-4 treated Ana-1 cells (P ≤ 0.05) (Fig. 2A). IL-10, another M2 marker gene, was up-regulated by IL-4, which was also found to be dose-dependently inhibited by treatment with CHA in Ana-1 cells (supplementary Fig. 2C). A similar pattern of iNOS mRNA levels was also observed in IL-4-treated RAW264.7 cells at 0.1 or 1 μM of CHA (P ≤ 0.05, P ≤ 0.01) (Fig. 2B). Furthermore, IL-4-induced CD206 expression was decreased by 10% at 2 μM of CHA in Ana-1 cells, but remained unchanged in RAW264.7 cells (Fig. 2C,D). These findings indicated that CHA effectively suppressed M2 phenotypic differentiation in Ana-1 macrophages.

### CHA regulated macrophage polarization by promoting STAT1 activation and suppressing STAT6 activation

**Figure 3:**
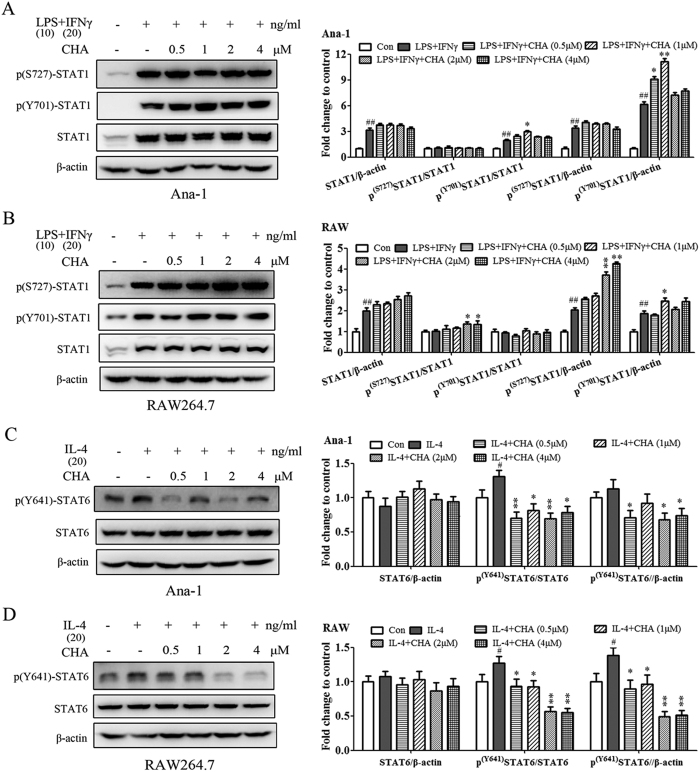
Effect of CHA on the STAT signaling pathways in macrophages. Ana-1 (**A**) and RAW264.7 (**B**) cells were treated with lipopolysaccharide (LPS) (10 ng/ml) and interferon (IFN)-γ (20 ng/ml) with or without different concentrations of CHA for 24 h. The expressions of Ser727-phosphorylated STAT1 (p-STAT1^ser727^), Tyr701-phosphorylated STAT1 (p-STAT1^Tyr701^) and STAT1were evaluated by a Western blot analysis. Ana-1 (**C**) and RAW264.7 (**D**) cells were treated with interleukin (IL)-4 (20 ng/ml) in the presence of DMSO or indicated concentrations of CHA for 24 h. The expressions of Tyr641-phosphorylated STAT6 (p-STAT6^Tyr641^) and STAT6 were evaluated by a Western blot analysis. The histogram bars represent three independent experiments. The data are presented as the mean ± SD. ^#^p-value < 0.05, ^##^p-value < 0.01 vs. Con. *p-value < 0.05, **p-value < 0.01 vs. LPS/IFNγ or IL-4.

We next sought to investigate the molecular mechanisms of CHA on macrophage polarization. Signal transducer and activator of transcription 1 (STAT1) and STAT6 signaling pathways play critical roles in the M1 and M2 macrophage polarization, respectively^36^,^37^. We detected the STAT1 and STAT6 activation after treatment with CHA in M1/M2 inducer stimulated-macrophage. As shown in Fig. 3A,B, the protein expressions of p(Ser727)-STAT1, p(Tyr701)-STAT1 and total STAT1 were significantly upregulated when Ana-1 and RAW264.7 cells were treated with M1-inducer (LPS/IFNγ) (P ≤ 0.01), and phosphor-STAT1 expression was further increased by the administration of CHA, as shown by significant increases in expressions of p(Tyr701)-STAT1 and p(Tyr701)-STAT1/total STAT1 at 1 μM of CHA in Ana-1 cells and by significant induction of p(Ser727)-STAT1 and p(Ser727)-STAT1/total STAT1 in RAW264.7 cells at high dose of CHA (2, 4 μM). Meanwhile, upregulations of p(Tyr641)-STAT6 and p(Tyr641)-STAT6/total STAT6 were observed when Ana-1 or RAW264.7 cells were treated with IL-4 for 24 h, which were significantly reduced by combination with CHA in a dose-dependent manner, particularly in RAW264.7 cells (P ≤ 0.05, P ≤ 0.01) (Fig. 3C,D). These results indicated that CHA could have a regulating effect on macrophage polarization mainly via STAT1 and STAT6 signaling pathways.

### CHA inhibited the tumor cell growth by affecting the interactions between macrophages and cancer cells

**Figure 4:**
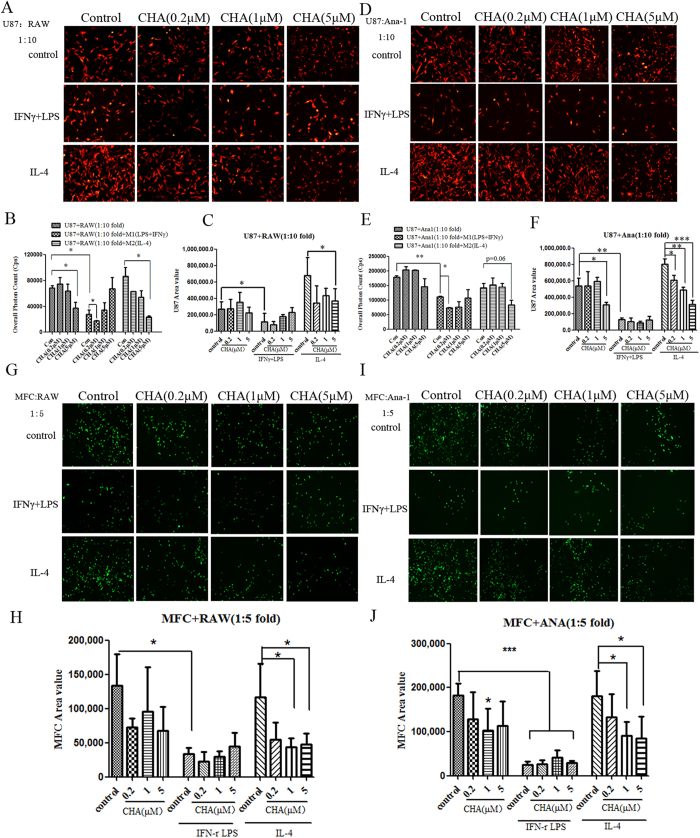
Effect of CHA on tumor cells growth in co-culture of tumor cells with macrophages. Tumor cells (1 × 10^3^) were mono-cultured in lipopolysaccharide (LPS) (10 ng/ml) and interferon (IFN)-γ (20 ng/ml) (M1 stimulator) or interleukin (IL)-4 (20 ng/ml) (M2 stimulator) alone, or in combination with indicated concentration of CHA for 48 h, or were co-cultured with M1 stimulator or M2 stimulator-treated macrophages in the presence of CHA for 48 h. U87-RFP-Luc glioma cells were co-cultured with RAW264.7 (**A**) or Ana-1 (**D**) cells, and then tumor cellular morphology was visualized by fluorescence microscopy. The proliferation of U87-RFP-Luc cell was represented as fluorescent area (**B**,**E**) or overall photon counts of cells (**C**,**F**) by using software of ImageProPlus and EnSpire Multimode Plate Reader, respectively. MFC-GFP forestomach cancer cells were co-cultured with RAW264.7 (**G**) or Ana-1 (**I**) cells. The morphology of MFC-GFP cells were visualized by fluorescence microscopy and the proliferation of MFC-GFP cells were assessed by overall fluorescent area of cells using ImageProPlus software (**H**,**J**). The histogram bars represent three independent experiments. The data are presented as the mean ± SD. *p-value < 0.05, **p-value < 0.01; as evaluated using Student’s t-test.

Considering that the highest concentration of CHA used in macrophages polarization did not directly affect the proliferation of tumor cell lines and rodent nerve cells including primary neurons and glial cell lines (CTX and BV2, etc) that could serve as origin of gliomas (Supplementary Fig. 1A–C), we examined whether CHA regulates the interactions between macrophages and brain cancer cells. The proliferation and morphological feature of U87 cells were almost unaffected by treatment with indicated concentrations of CHA, IFNγ/LPS (M1 inducer), or IL-4 (M2 inducer) alone (Supplementary Fig. 3). In the condition of co-culture experiments, the proliferation of U87 cells was increased by co-cultured with M2 inducer-treated macrophages cell lines (RAW264.7 and Ana-1), and this pro-tumor function of the M2 phenotypic macrophages was significantly suppressed by CHA treatment (Fig. 4A–F). Furthermore, we found that the growth of U87 cells was significantly decreased in co-cultured with M1 inducer-treated macrophages (P ≤ 0.05), and the anti-tumor effect of CHA under the assay conditions used was performance in the induction of U87 cells to be as an apoptotic-like shape (Fig. 4A–F). A similar phenomenon was observed when murine forestomach cancer cell MFC-GFP was co-cultured with M1/M2-inducer stimulated macrophages, treatment with 1 μM or 5 μM CHA also decreased the stimulatory role of M2 phenotypic macrophage on tumor growth (Fig. 4G–J). These data indicated that CHA exhibited antitumor effect through intervention of the cell-cell interactions between macrophages and cancer cells.

### CHA inhibited glioma growth in murine model

**Figure 5:**
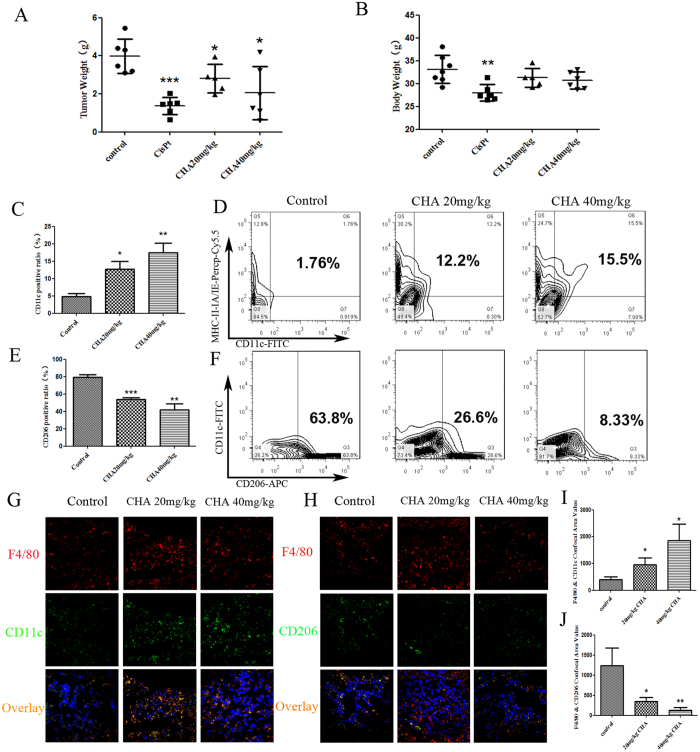
Effect of CHA on glioblastoma progression in G422 xenograft model. ICR mice were injected subcutaneously in the flank with G422 glioma cells in and were treated with CHA (20 mg/kg or 40 mg/kg per day) for 14 days or cisplatinum (CPT) (6 mg/kg per week). The tumor weight (**A**) and body weight (**B**) in individual mice were detected. The infiltration of M1 macrophages (**C**) in the tumor tissues were evaluated using flow cytometry. The percentage of CD11c, MHC-II IA/IE and F4/80 positive macrophages was presented in the histogram (**D**). The infiltration of M2 macrophages (**E**) in the tumor tissues were evaluated using flow cytometry. The percentage of CD206 and F4/80 positive macrophages was presented in the histogram (**F**). Furthermore, M1 macrophages infiltrating in tumor sections were visualized by immunofluorescence staining of F4/80 and CD11c (**G**). M2 macrophages infiltrating in tumor sections were visualized by immunofluorescence staining of F4/80 and CD206 (**H**). The F4/80^+^CD11c^+^ area (**I**) and the F4/80^+^CD206^+^ area (**J**) (% of F4/80^+^ tumor area) were qualified as area value of overlapping fields. Five random fields from sections of each mouse tumor xenograft were examined. The data are presented as the mean ± SD. *p-value < 0.05, **p-value < 0.01 vs. control.

We next evaluated the anti-cancer effect of CHA using an *in vivo* xenograft G422 glioma murine model. As shown in Fig. 5A,B, CHA was able to inhibit the tumor growth of G422-bearing mice in a dose-dependent manner without loss of body weight. Tumor inhibition rates were 30% and 48% in the groups treated with 20 mg/kg and 40 mg/kg CHA, respectively (P ≤ 0.05). In addition, flow cytometry analysis was performed to investigate the effects of different phenotypic macrophages infiltrating in tumor tissue after the administration of CHA. Triple immune-staining of CD11c, MHC-II IA/IE and F4/80 results showed that CHA treated G422-bearing mice contained more CD11c^+^ and MHC-II IA/IE^+^ double positive cells in F4/80^+^ macrophages infiltrating in tumor tissue than the control group (Fig. 5C,D). Co-immunostaining of CD206, CD11c and F4/80 showed that the percentages of macrophages expressed CD206 in tumor tissue of CHA-treated mice were fewer than the control group (26.6% in 20 mg/kg group; 8.33% in 40 mg/kg group Vs 63.8% in control group) (Fig. 5E,F). Furthermore, the results from immunofluorescence staining further confirmed the above findings, M1-type macrophages that showed positive staining with antibodies to CD11c and F4/80 were increased in tumor sections from CHA-treated mice (Fig. 5G,I), while M2 macrophages infiltrating into tumor sections that immunostaining with CD206 and F4/80 were significantly reduced by CHA treatment (P ≤ 0.05, P ≤ 0.01) (Fig. 5H,J). Taken together, these results indicated that treatment with CHA significantly reduced the infiltrating M2-type macrophages but increased the M1-type macrophages accumulation in tumor tissue from G422-bearing mice. In addition, in G422 orthotopic glioma model, we found that the slices of intracranial tumor and tumor volume visualized in magnetic resonance imaging (MRI) were decreased after CHA administration (Supplementary Fig. 4A,B).

## Discussion

Although CHA has been in phase I clinical trials for multiple types of cancer therapy, little has been reported on the anti-tumor effect of CHA on glioma through modulating macrophage phenotype differentiation. Herein we, for the first time, demonstrated that CHA treatment inhibited anti-inflammatory sate and induced activated macrophage to antitumor pro-inflammatory phenotype, consistent with the inhibition of glioma growth *in vitro* and *in vivo*.

The majority of previous studies on CHA have been focused on its direct toxicity to tumor cells. Some research have shown that CHA inhibited tumor growth and tumor angiogenesis in several types of malignancy including glioblastoma through the inhibition of tyrosinase and matrix metalloproteinase (MMP)-9 or by induction of p38 mitogen-activated protein kinase-dependent apoptosis^29^,^30^. In these studies, the effective dose of CHA was more than 10 μM, or reached to 50 μM. In our study, we found that CHA almost did not exhibit direct toxicity towards either of various human cancer cell lines at concentrations lower than 10 μM which is the highest plasma concentration of CHA that achieved in clinical tumor patients following intraperitoneal administration of 3 mg/kg (Supplementary Fig. 1A). Therefore, it is likely that CHA inhibits the growth of glioma predominately through modulating the tumor microenviroment in our study.

In malignant gliomas, there is M2-polarization of microglia acquiring immunosuppressive and tumor-supportive. Inhibition of macrophage differentiation to the M2 phenotype has been proposed to increase the tumor-associated immune response in patients with glioblastoma^38^. The findings of this study showed that treatment with CHA in glioma-bearing mice inhibited the distribution of M2 type macrophages (F4/80^+^/CD206^+^ double labeled cells), consistent with the reduction of tumor weight observed in these mice. Meanwhile, we cannot exclude that, at least *in vivo*, the CHA-induced phenotype switch to M1 (F4/80^+^ and CD11c^+^/MHC-IIIA-IE^+^) could be secondary effect of the drug on glioma. This is in line with the results *in vitro*, the up-regulation of anti-inflammatory M2 makers Arg1 and CD206 induced by IL-4 were all reduced upon CHA treatment. And, CHA further increased levels of the pro-inflammatory M1 markers, iNOS, CD11c and MHCIIIA-IE, in the present of LPS/IFNγ treatment.

Increasing evidences have shown that M1-polarized macrophages are likely to play an anti-tumoral response, while M2-polarized macrophages have been associated with malignant tumor growth, angiogenesis, migration and invasion^39^,^40^. Such support is new provided by our co-culture experiments, LPS/IFNγ-treated macrophages (M1-type) could inhibit the growth of U87 glioma cells and MFC breast cancer cells, and CHA was able to affect the interaction of M1 macrophages with glioma cells and induced glioma cells toward an apoptotic-like round shape. In contrast, IL-4-treated macrophages (M2-type) promoted the growth of tumor cells, and lower dose (1 μM) of CHA could significantly inhibit the M2 macrophages-induced proliferation of glioma and breast cancer cells. Taken together, these data support the hypothesis that the *in vivo* and *in vitro* antitumor effect of CHA depends on the macrophage polarization. We suggested that CHA is not only a potent inhibitor of M2 activation but also an enhancer for M1 activation.

Many studies have revealed the detailed signaling mechanisms of macrophage differentiation. Our finding showed that CHA regulated LPS/IFNγ- and IL-4 responsive genes through promoting STAT1 activation and inhibiting STAT6 activation. In addition to the STAT family, NF-κB, the nuclear receptor PPAR-γ and the CREB-C/EBP axis all participate in macrophage polarization^36^,^41^. Previously, CHA has also been reported to exert anti-inflammatory activities in LPS-treated RAW264.7 cells via attenuating the activation of inflammatory signaling pathways NF-κB and JNK/AP-1^27^,^42^. In our preliminary data, NF-κB activation was not changed by CHA (data not shown). There is still some controversy in the regulatory role of NF-κB on macrophage polarization. Some paper reported that NF-κB signaling mediated M1 phenotype differentiation. NF-κB drives the transcription of many pro-inflammatory genes in macrophages, such as IL-12p40, NOS2 and STAT1/2^43^. S. Gao and S. Iwanowycz, *et al*. have reported that several Chinese herb-derived antioxidants, such as Curcumin and Emodin, could suppress the excessive response of M1 macrophages via blockade of NF-κB signaling pathway^20^,^44^,^45^,^46^,^47^. But others indicated that NF-κB activation plays an important role in macrophage differentiation toward the M2 phenotype. For instance, Y. Fujiwara and colleagues reported that corosolic acid suppresses the M2 polarization of macrophages and tumor cell proliferation by inhibiting both STAT3 and NF-κB activation^48^. Furthermore, the direct molecular targets of CHA in macrophages in the context of cancer warrant further identification.

In conclusion, we demonstrated that CHA inhibited the growth of glioblastoma *in vivo* and *in vitro* at least partially through inhibiting M2 polarization and further skewing macrophage polarization away from the M2- to M1-like phenotype via promotion of STAT1 activation and inhibition of STAT6 activation, respectively. Overall these findings indicated CHA can be used to mediate macrophage polarization to carry out its anti-glioma function.

## Materials and Methods

### Cells and cell culture conditions

U87 human glioma cell line stable expressed red fluorescence protein (RFP) and luciferase (U87-RFP-Luc) was purchased from the Keyuandi, Biological Technology Development Co., Ltd. (Shanghai, China). MFC mouse forestomach cancer cell line labeled with green fluorescence protein (MFC-GFP), and mouse macrophage cell lines, RAW264.7 and Ana-1, were purchased from the Institute of Medical Sciences, Peking Union Medical College (Beijing, China). The cells were cultured in RPMI 1640 medium and Dulbecco’s modified Eagle’s medium (DMEM), respectively, supplemented with 10% fetal bovine serum (FBS) (Gibco, ThermoFisher Scientific, USA) and 100 units/ml penicillin, and 100 units/ml streptomycin in a humidified atmosphere of 5% CO_2_ at 37 °C. All protocols using human cell lines were approved by the Research Ethics Committee of the Institute of Materia Medica, Chinese Academy of Medical Sciences & Peking Union Medical College, and conducted in accordance with the approved guidelines for safety requirements. M1 macrophages were polarized by stimulating with 10 ng/ml lipopolysaccharide (LPS) (Sigma-Aldrich) and 20 ng/ml interferon (IFN)-γ (Peprotech). M2 macrophages were polarized by stimulating with 20 ng/ml interleukin (IL)-4 (Peprotech).

### Anti-cancer drugs

Chlorogenic acid (CHA) was provided by Jiuzhang Biochemical Engineering Science and Technology Development Co., Ltd. (Chengdu, Sichuan, China), and dissolved in dimethyl sulfoxide (DMSO) at the appropriate concentrations.

### RNA extraction and quantitative real-time polymerase chain reaction (qRT-PCR)

Total RNA was extracted from macrophages cell lines using TRIzol reagents following the manufacturer’s recommended procedures (Invitrogen, Carlsbad, CA, USA). The total RNA was reverse-transcribed to cDNA using a ReverTra Ace quantitative reverse transcription-polymerase chain reaction (qRT-PCR) Kit (Toyobo, Osaka, Japan). Real-time PCR with the ABI PRISM 7900 sequence detection system (Perkin-Elmer, Branchburg, NJ, USA) was performed using the SYBR Green PCR Master Mix (Cat. QPK-201, Toyobo, Osaka, Japan). The expression of mRNAs was normalized to GAPDH. Data are presented as relative quantification based on the calculation of 2^−ΔΔCt^. All primer sequences used for PCR were shown in Supplementary Dataset.

### Protein extraction and western blotting assay

Ana1 and RAW264.7 cells were harvested and homogenized with RIPA buffer for Western blot analysis, as previously described^49^. Briefly, equal amounts of proteins (~30 μg) were separated by SDS-polyacrylamide gel and transferred onto a polyvinylidene fluoride (PVDF) membrane (Millipore, Bedford, MA, USA). Membranes were blocked in 5% dried milk powder and then incubated with primary antibodies overnight at 4 °C. The primary antibodies were phosphor-STAT1^ser727^, phosphor-STAT1^Tyr701^, STAT1, phosphor-STAT6^Tyr641^, STAT6 (Cat. #8826, #9167, #14994, #9361 and #5397, Cell Signaling Technology, USA). After incubation with appropriate horseradish peroxidase (HRP)-conjugated secondary antibodies, bound proteins were visualized by enhanced chemiluminescence (ECL) (Applygen Technologies Inc, Beijing, China) and detected using ImageQuant LAS 4000 (GE Healthcare, Piscataway, NJ, USA). Blots were performed in triplicate. The relative protein levels were calculated based on β-actin (Cat. #sc-47778, Santa Cruz Biotechnology Inc.) as the loading control.

### Co-culture assays of tumor cells with macrophages

U87-RFP-Luc human glioma cells or MFC-GFP mouse forestomach cancer cells were monocultured or directly co-cultured with RAW264.7 or Ana-1 macrophages at a density of 1:10 or 1:5 in a 96-well plate. After being cultured overnight, the cells were treated with 0.2, 1 and 5 μM of CHA alone, or M1 inducer (10 ng/ml, LPS; 20 ng/ml, IFNγ), or M2 inducer (20 ng/ml, IL-4) with or without CHA for 48 h. Then, the morphological features of the fluorescence-labeled tumor cells were examined under fluorescence microscopy, and the bioluminescence of glioma cells was detected after incubation of 15 μg/ml luciferase for 5 min using an EnSpire Multimode Plate Reader (PerkinElmer, Inc, USA).

### Mouse G422 glioma cell xenograft model

Female ICR mice (age: 6~8 weeks; weight: 18~22 g) were purchased from the Institute of Experimental Animal Research, Peking Union Medical College (Beijing, China). Mouse G422 glioma cells (5 × 10^5^) in 0.2 ml of phosphate-buffered saline (PBS) were injected subcutaneously into the right flank of the ICR mice. The following day after tumor implantation, mice were randomly distributed into four groups (n = 6 per group). CHA or vehicle (normal saline) was administered intraperitoneally at 20 mg/kg or 40 mg/kg, daily for two weeks. The positive control group was given cisplatinum (CPT) (6 mg/kg) by intraperitoneal injection once per week. At the end of the experiments, mice were sacrificed, and tumors were removed, measured, and analyzed. For the intracranial tumor experiments, ICR mice were anesthetized and stereotaxically injected with 2 × 10^5^ G422 cells in 5 μL PBS, 2 mm right and 1 mm anterior to the bregma in the striatum at 3 mm depth with a 10 μL Hamylton syringe needle. After administration of CHA for 9 days, the anatomical images of the intracranial tumors were visualized by small animal MRI scanner (Pharma Scan 70/16 US, Bruker, Germany). All animal experiments were approved by the Ethics Committee for Animal Experiments of the Institute of Materia Medica, Chinese Academy of Medical Sciences & Peking Union Medical College and conducted in accordance with the Guidelines for Animal Experiments of Peking Union Medical College.

### Macrophage marker expression by flow cytometry

RAW264.7 and Ana-1 macrophage cell lines were harvested and blocked with 3% BSA for 45 min, and then were stained with Alexa Flour 488 anti-mouse CD11c antibody, PerCP-Cy5.5-conjugated anti-mouse MHC Class II subunits IA-IE antibody, allophycocyanin (APC)-conjugated anti-mouse CD206 antibody or APC/Cy7-conjugated anti-mouse F4/80 antibody (Cat. #117311, #107626, #141708 and #123118, BioLegend, San Diego, CA, USA). For the analysis of macrophage marker expression in xenograft tumor tissue, tumor was minced into small pieces and incubated with 1 mg/mL collagenase Type IV and 300 U/mL DNaseI (Cat. #C5138 and #D5025, Sigma Aldrich) at 37 °C for 30 min. The tissue suspension was passed through a 70 μm cell strainer (BD Biosciences, Bedford, MA, USA), and was stained with above-mentioned fluorescein-labeled antibodies including anti-CD11c, anti-MHC II subunits IA-IE, anti-CD206 and anti-F4/80. The stained cell samples were analysed using a FACSVerse mechine (Becton Dickinson, San Diego, CA, USA). The proportion of positively-stained cells was analyzed with the FlowJo software package (Tree Star, Ashland, OR, USA).

### Immunofluorescence

Tumor tissues from G422-bearing mice were immediately frozen in OCT embedding medium, and were cut at 8 μm thicknesses. The M1-type macrophage markers were detected by incubation with the primary monoclonal antibodies to rat anti-F4/80 and mouse anti-CD11c (Cat. #ab6640 and #ab11029, Abcam, Cambridge, MA, USA). For the analysis of M2 phenotypic macrophage, frozen sections were immunostained with rat anti-F4/80 and rabbit anti-CD206 antibodies (Cat. #ab6640 and #ab64693, Abcam, Cambridge, MA, USA). The sections were subsequently treated with appropriate fluorescein-labeled secondary antibodies to anti-rat Alexa Fluor 647 dye (red) and anti-mouse Alexa Fluor 488 dye (green) or anti-rabbit Alexa Fluor 488 dye (green) (Invitrogen-Molecular Probes, Carlsbad, CA, USA). Slides were counterstained with the blue nuclear counterstain, 4′,6-diamidino-2-phenylindole (DAPI) for 5 min and then imaged using an Olympus FV1000 confocal microscope. For quantitative analysis, the number of positive cells was analyzed in six random fields of view per section using an image analysis program Image Pro-Plus, version 6.0 (Media Cybernetics, Rockville, MD, USA).

Statistics. All data are representative of two or three independent experiments. The data are expressed as the means ± standard deviation (S.D). Comparisons used two-tailed Student’s t-test or ANOVA analysis as appropriate. P-values of < 0.05 were considered to be statistically significant difference.

## Additional Information

**How to cite this article**: Xue, N. *et al*. Chlorogenic acid inhibits glioblastoma growth through repolarizating macrophage from M2 to M1 phenotype. *Sci. Rep.* **7**, 39011; doi: 10.1038/srep39011 (2017).

**Publisher's note:** Springer Nature remains neutral with regard to jurisdictional claims in published maps and institutional affiliations.

## Supplementary Material

Supplementary Dataset 1
