# Supplementary material for: Chlorogenic acid inhibits glioblastoma growth through repolarizating macrophage from M2 to M1 phenotype
Source: Sci Rep. 2017 Jan 3;7:39011. doi: 10.1038/srep39011 (PMC5206721; doi:10.1038/srep39011)
Supplement: Supplementary Dataset 1 [file srep39011-s1.doc]

**Chlorogenic acid inhibits glioblastoma growth through repolarizating macrophage from M2 to M1 phenotype**

Nina Xue^1,*^, Qin Zhou^1,*^, Ming Ji^1^, Jing Jin^1^, Fangfang Lai^1^, Ju Chen^1^, Mengtian Zhang^2^, Jing Jia^2^, Huarong Yang^2^, Jie Zhang^2^, Wenbin Li^3^ & Jiandong Jiang^1^ & Xiaoguang Chen^1^

^1^State Key Laboratory of Bioactive Substances and Functions of Natural Medicines, Institute of Materia Medica, Chinese Academy of Medical Sciences and Peking Union Medical College, Beijing 100050, China

^2^Jiuzhang Biochemical Engineering Science and Technology Development Co., Ltd., Chengdu, Sichuan 610041, China

^3^Department of Glioma, Beijing Shijitan Hospital, Capital Medical University, Beijing 100038, China

^*^The first two authors contributed equally to this work！

Corresponding authors:

Xiaoguang Chen, Jiandong Jiang and Wenbin Li are willing to handle correspondence at all stages of refereeing and publication, also post-publication.

*E-mail addresses: [chxg@imm.ac.cn](mailto:chxg@imm.ac.cn) (X-G Chen); [jiangjd@imm.ac.cn](mailto:jiangjd@imm.ac.cn) (J-D Jiang); [liwenbin@ccmu.edu.cn](mailto:liwenbin@ccmu.edu.cn) (W-B Li)

Telephone number: +86(10) 63165207

Address: No.1 Xian Nong Tan Street, Beijing, China

Zip Code: 100050

**Table 1．The primer sequences used in Real-time RT-PCR analysis.**

| Gene | Strands | Sequence |
| --- | --- | --- |
| iNOS | Forward | 5’- TCAGCTACGCCTTCAACACC -3’ |
|  | Reverse | 5’- TTCCCAAATGTGCTTGTCACC-3’ |
| Arg1 | Forward | 5’- ATCAACACTCCCCTGACAACC-3’ |
|  | Reverse | 5’- CGCAAGCCAATGTACACGAT-3’ |
| IL10 | Forward | 5’- ACCTGCTCCACTGCCTTGCT-3’ |
|  | Reverse | 5’- GGTTGCCAAGCCTTATCGGA-3’ |
| IL12p40 | Forward | 5’- AGCAGTAGCAGTTCCCCTGA-3’ |
|  | Reverse | 5’- AGTCCCTTTGGTCCAGTGTG-3’ |
| GAPDH | Forward | 5’- CATCTTCCAGGAGCGAGACCC-3’ |
|  | Reverse | 5’- AGACACCAGTAGACTCCACGACA-3’ |


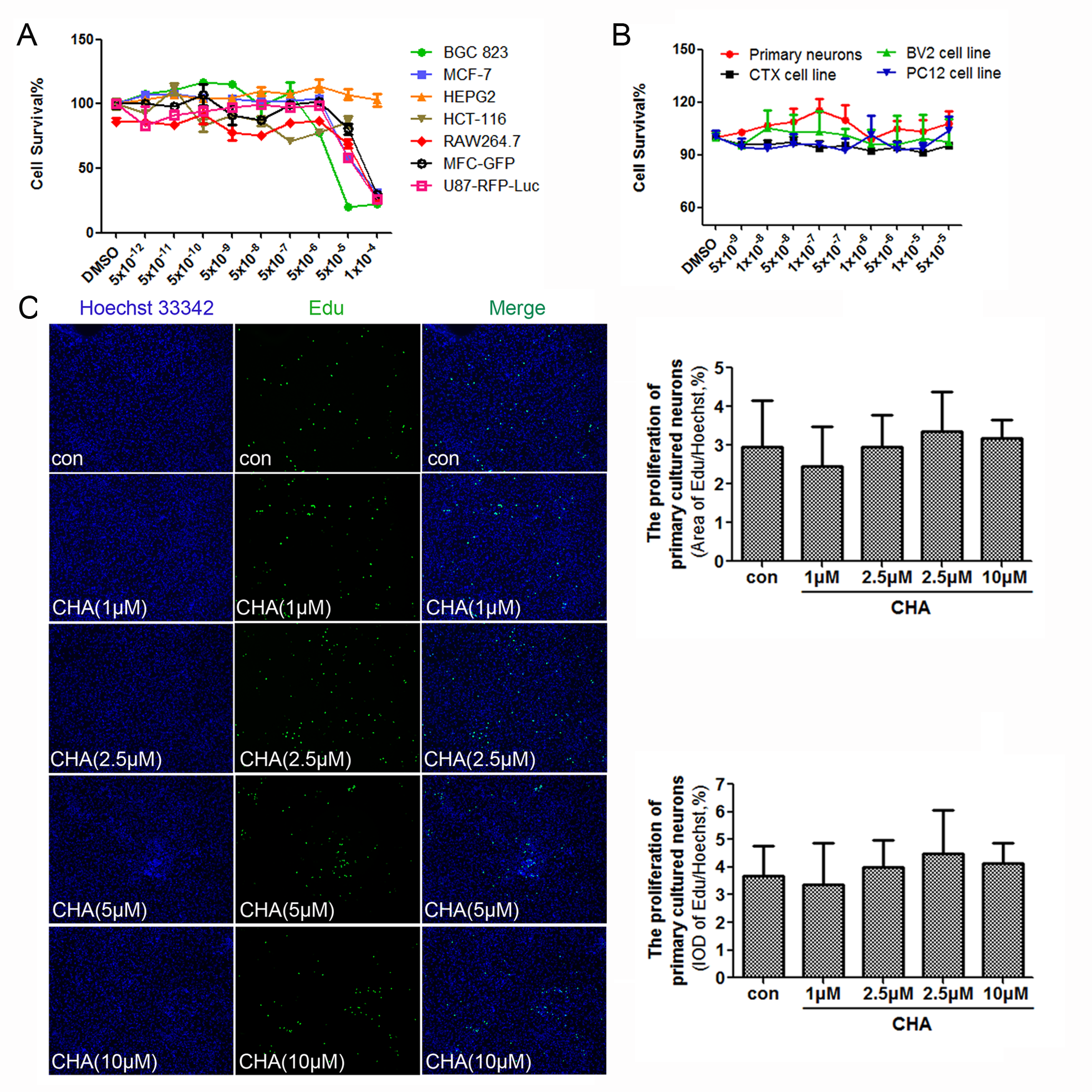


Figure S1. The effect of CHA on the proliferation of tumor, macrophage and nerve cells. Various tumor cell lines and macrophage cells were treated with different concentration of CHA for 96 h and subjected to MTT assays (A). Primary neurons from neonatal SD rats, glial cell lines (CTX and BV2) and PC12 cell lines were treated with different concentration of CHA for 96 h and subjected to MTT assays (**B**). Primary neurons were treated with indicated concentration of CHA and subjected to Edu immuofluorescence staining. The percentage of proliferative primary neurons was calculated by the Area or IOD of Edu/Hoechst and was presented in the histogram (**C**).


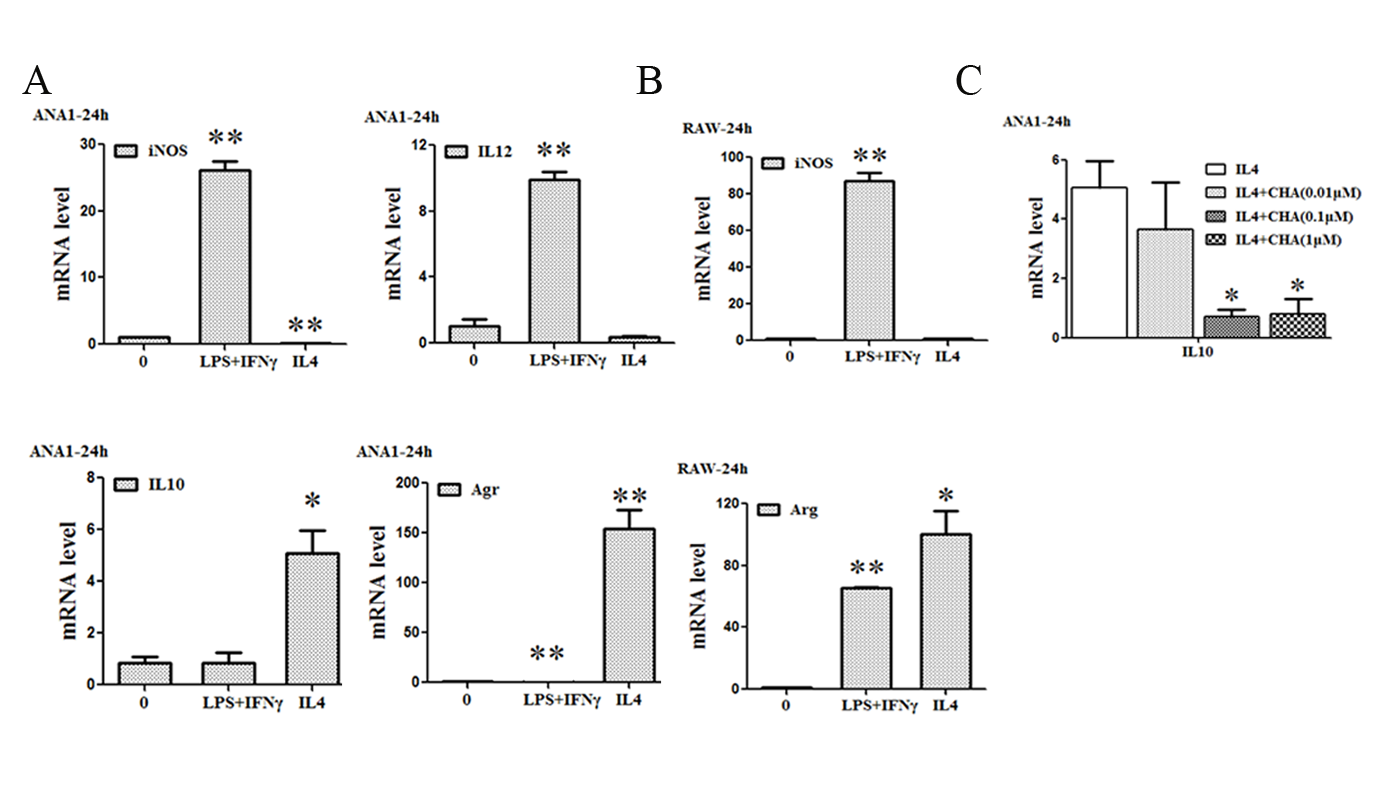


Figure S2. *iNOS*, *Arg1*, *IL10* and *IL12p40* mRNA levels after treated with lipopolysaccharide (LPS) and interferon (IFN)-γ or interleukin (IL)-4 with or without CHA. Real-time RT-PCR was carried out to detect the mRNA levels of *iNOS*, *Arg1*, *IL10* and *IL12p40* genes after treated with LPS (10 ng/ml) and IFNγ (20 ng/ml) or interleukin (IL)-4 (20 ng/ml) in Ana-1 (**A**) and RAW264.7 cells (**B**). *IL10* mRNA levels in Ana-1 cells after exposure to IL-4 (20 ng/ml) alone, or combination with indicated concentrations of CHA for 24 h (**C**). The histogram bars represent three independent experiments. The data are presented as the mean ± SD. *p-value<0.05, ** p-value<0.01 vs. control or IL-4.

**
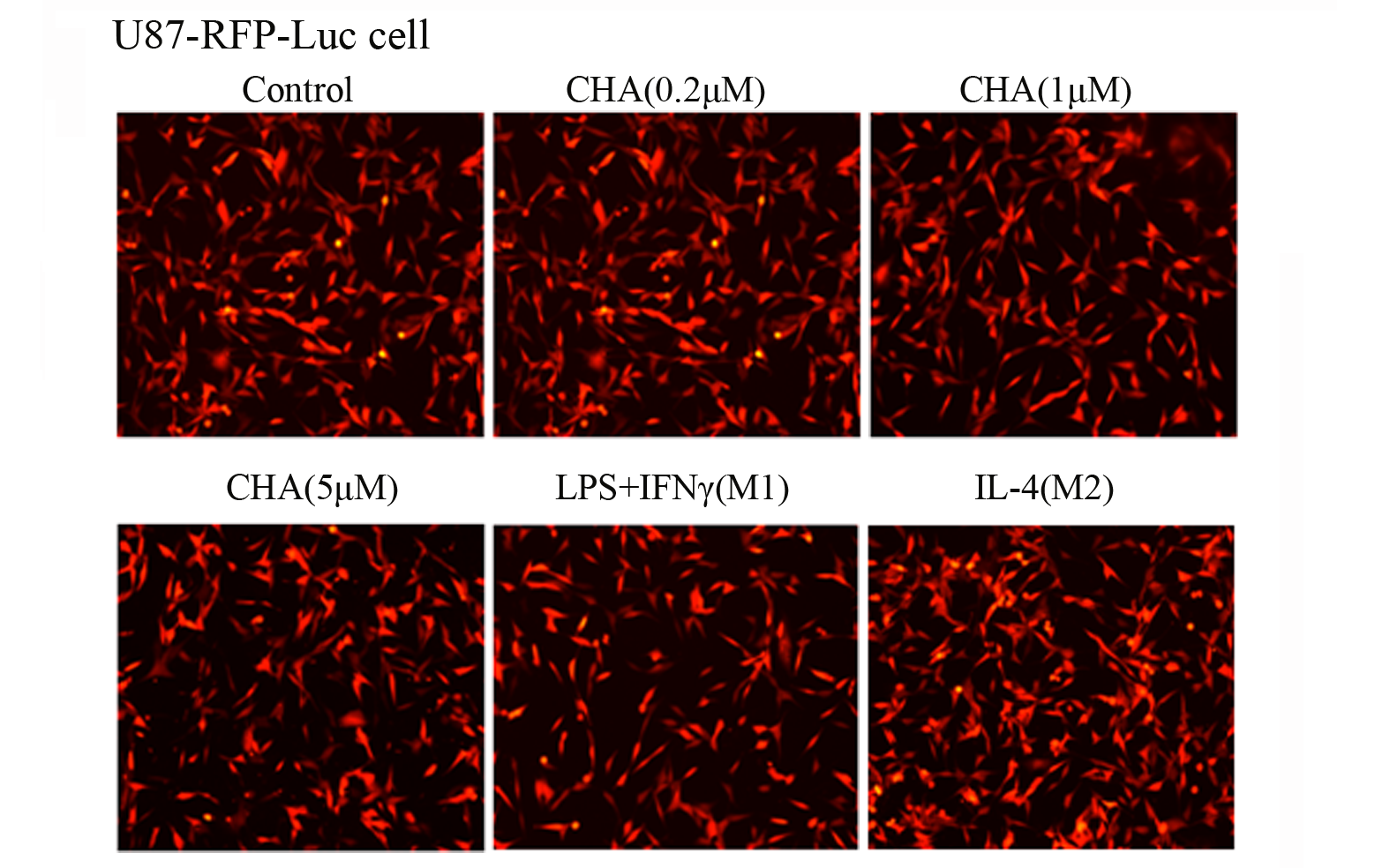
**

Figure S3. The effect of CHA and M1/M1 inducer on the proliferation of U87-RFP-Luc brain tumor cells. U87-RFP-Luc glioma cells were treated different concentration of CHA or M1/M2 stimulators, IFNγ/LPS and IL-4. The morphological feature of tumor cells was examined under fluorescence microscopy.


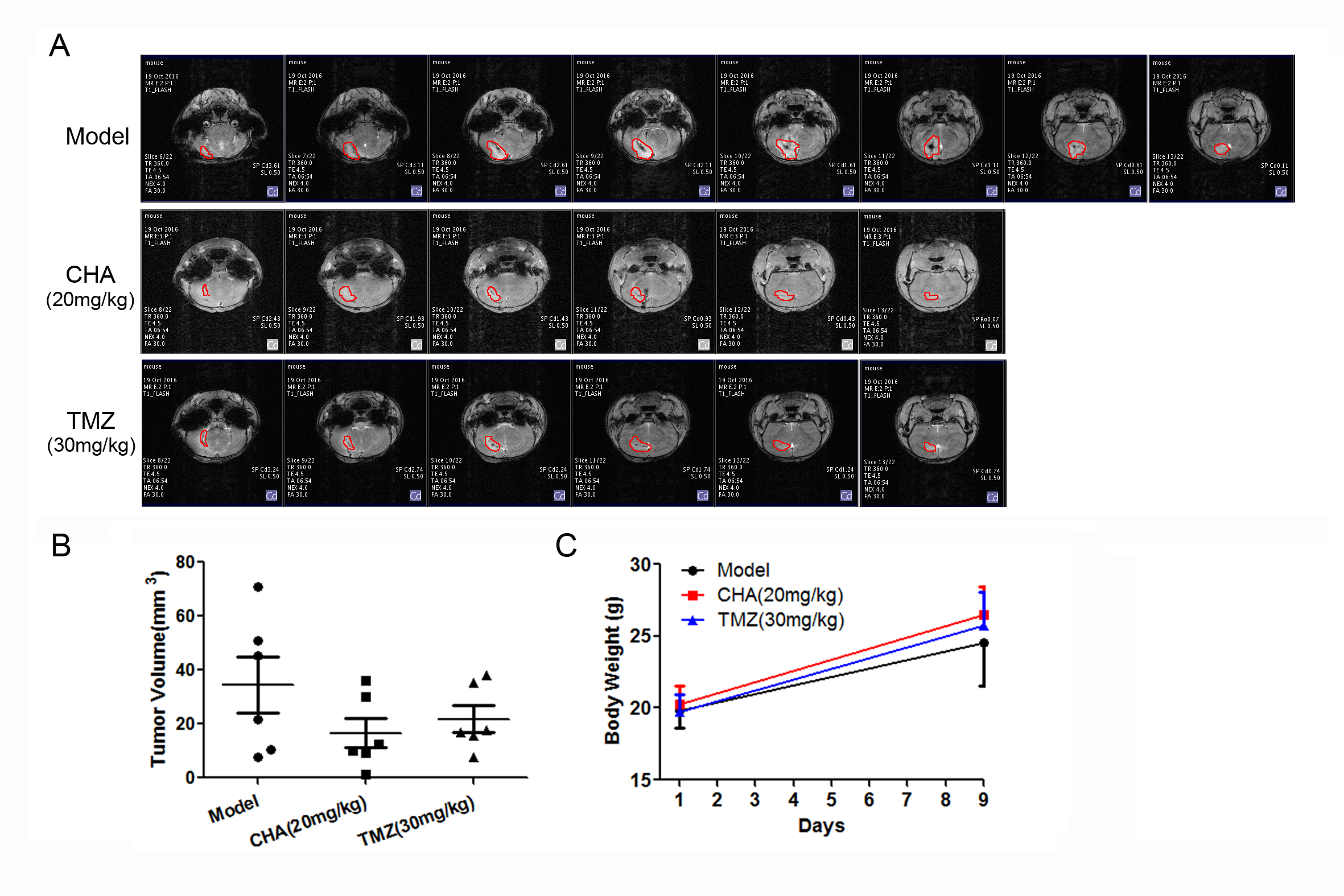
**Figure** S4**: Effect of CHA on glioblastoma progression in G422 orthotopic glioma mode**l. ICR mice were anesthetized and stereotaxically injected with 2×10^5^ G422 cells in 5μL PBS, 2 mm right and 1 mm anterior to the bregma in the striatum at 3 mm depth with a 10µL Hamylton syringe needle. After daily i.p. injected CHA (20 mg/kg) for 9 days, the anatomical images of the intracranial tumors were visualized by small animal MRI scanner. The representative image of intracranial tumors in each group was shown (**A**). The tumor volume of individual mice was analysis using soft of Dicom and calculated using the formula V_M_=Length(max)×Width×Thickness (**B**). Body weight in each group were presented as the mean ± SD (**C**).
